# Enhanced azo dyes decolorization in bioaugmented floating treatment wetlands using *Typha domingensis* and *Bacillus* sp.

**DOI:** 10.3389/fmicb.2026.1848974

**Published:** 2026-07-13

**Authors:** Sadia Zahid, Kaneez Fatima, Muhammad Ali, Mohsin javed, Hina Ibraheem, Maryam Afzal, Mohammed Abohashrh, Shahid Iqbal, Sajid Mahmood, Ibrahim Jafri, Hala Mohamed Abdelmigid, Abd-ElAziem Farouk, Amal Alyamani

**Affiliations:** 1 Department of Life Sciences, School of Science, University of Management and Technology (UMT), Lahore, Pakistan; 2 Department of Chemistry, School of Science, University of Management and Technology (UMT), Lahore, Pakistan; 3 School of Chemical Engineering, Aalto University, Espoo, Finland; 4 Department of Basic Medical Sciences, College of Applied Medical Sciences, King Khalid University, Abha, Saudi Arabia; 5 Department of Chemical and Environmental Engineering, University of Nottingham Ningbo China, Ningbo, China; 6 Low Dimensional Materials Research Center at Khazar University, Baku, Azerbaijan; 7 Department of Biotechnology, College of Science, Taif University, Taif, Saudi Arabia

**Keywords:** azo dye de-coloration, *Bacillus* sp., bioaugmentation, floating treatment wetlands, *Typha domingensis*

## Abstract

The present study evaluated bioaugmented floating treatment wetlands (FTWs) for the effective remediation of textile waste enriched with the azo dyes Synozol Red K3 BS (SR), Synozol Yellow K3 RS, and Synozol Ultra Black DR (SB). Six strains of bacteria (SZ1-SZ6) were isolated, characterized and tested for the ability to decolorize dyes. Of these, the SZ1 strain, *Bacillus* sp., was observed to have a maximum removal rate of 92% for SR, 89.96% for SY and 54% for SB dyes in shake flask assays. The performance of SZ1 was further optimized under different physicochemical conditions, pH (1, 3, 5, 7, 9, 11, and 13), incubation temperature (30–40°C), incubation days (1, 3, 5, and 7), and inoculum size (1, 2, 3, and 4%). The results indicated that optimum conditions for maximum decolorization were at pH 7, temperature 37°C and 2% inoculum size. SZ1 was then optimized and further introduced in FTWs vegetated with Typha domingensis. Bioaugmented FTWs (T. domingensis + *Bacillus* sp.) achieved significantly more dye removal up to 95% compared with FTWs with only plants or bacteria. Moreover, growth parameters (root and shoot length) were improved in bioaugmented systems indicating reduced toxicity. The results demonstrated that *Bacillus* Sp. and T. domingensis were complementary effective in the performance of FTWs to treat the dye-contaminated water, which provided an environmentally friendly FTW-based remediation solution as compared to the conventional one.

## Graphical Abstract

**Figure:**
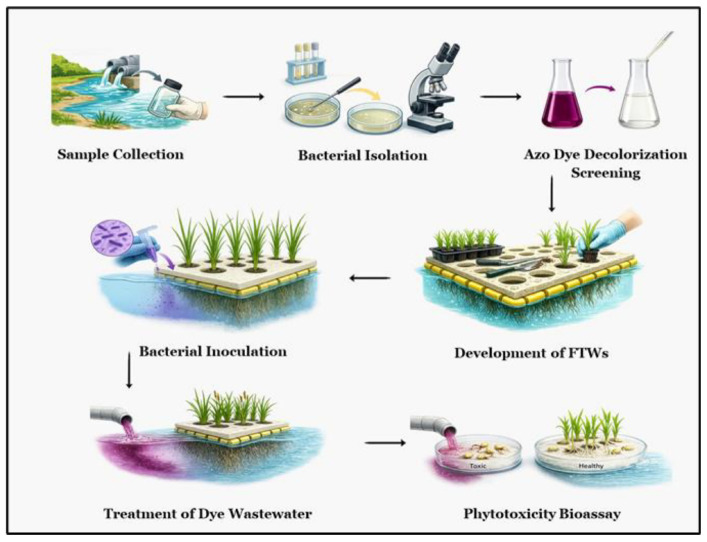
This study demonstrated that bioaugmented floating treatment wetlands (FTWs) integrated with *T. domingensis* and *Bacillus* sp.

## Introduction

The textile industry is a dynamic component of the economy of most countries, including Pakistan, and is an important source of employment, industrial output, and export earnings (Memon et al., 2020; Sebastian et al., 2026). Although the industry has economic benefits, it poses substantial environmental challenges as a consequence of significant amounts of untreated effluents discharged, including synthetic dyes, mostly azo dyes (Goswami et al., 2024). The annual production of synthetic dyes is expected to be 700,000 tons, which equals a total of around 280,000 tons of untreated dye compounds entering the surface waters of the world, where they can be found (Islam et al., 2025; Sebastian et al., 2026). These dyes lead to decreased light penetration, increased biological oxygen demand (BOD) and chemical oxygen demand (COD), disruption of aquatic photosynthesis, and even the release of toxic or carcinogenic aromatic amines during degradation (Sreedharan et al., 2021; Jahan and Singh, 2023). The treatment of dyes in industrial effluents by physicochemical methods has been used for many years. The methods used are coagulation, flocculation, advanced oxidation processes, and membrane filtration (Kusworo et al., 2024).

Such techniques, however, come with some limitations, such as higher operational expenses, energy consumption, and the generation of secondary waste, or sludge, which needs more treatment and disposal (Tripathi et al., 2023; Lotha et al., 2024). Biological treatments provide a promising, cost-effective, and environmentally friendly energy solution in comparison (Liu et al., 2024; Li et al., 2026; Luo et al., 2026; Wadhawan and Gupta, 2025). Bioremediation is one of the biological approaches which involves the use of living organisms, such as microorganisms and plants, and has been identified as a cost-effective and sustainable solution (Mazhar and Saleem, 2024; Tabassum et al., 2022). Azo dyes can be biotransformed by the action of microorganisms using enzymatic processes such as manganese peroxidase, lignin peroxidase, azo reductases, and laccases or by biosorption onto their cell walls (Dey and Mandal, 2025; Afoma et al., 2026; Rajendran et al., 2025). The removal of dyes by microorganisms is complemented by aquatic plants by the process of rhizodegradation or phytodegradation. The plant roots exude a number of substances that enhance the microbial activity in the rhizosphere and plant peroxidases and laccases may directly transform/degrade dye molecules (Balamurugan et al., 2025; Gayathiri et al., 2022). Therefore, the synergistic effect of the plant and microorganism is responsible for the FTWs which is a promising natural solution to treat dye polluted water.

FTWs are designed systems that use aquatic plants atop buoyant mats that let the roots of the plants hang into the water to replicate natural wetlands. The roots broaden the surface area available to the microorganisms to colonize, allowing them to form a symbiotic relationship where plants and microorganisms work together to mineralize and detoxify pollutants (Liu et al., 2024; Mao et al., 2024). Recent studies have shown that the use of dye-decolorizing bacteria (DDs) can greatly enhance the performance of FTWs (Nawaz et al., 2020; Patel et al., 2025; Fajri et al., 2025; Pandey et al., 2025). In this context, the present work aims to isolate and select an indigenous bacterial strain capable of decolorizing industrially relevant azo dyes. The selected strain was applied in FTWs vegetated with the native wetland plant *Typha domingensis* to evaluate enhanced remediation potential. To the best of our knowledge, unlike previous studies that have largely focused on single-model dyes, this study investigates the remediation of multiple commercially relevant azo dyes (Synozol red, yellow, and black), thereby better representing the complexity of textile effluents.

## Experimental section

### Reactive dyes

Three azo dyes, Synozol Red K3 BS (SR, C.I. Reactive Red 195), Synozol Yellow K3 RS (SY, C.I. Reactive Yellow 145), and Synozol Ultra Black DR (SB, C.I. Reactive Black 5), were purchased from a chemical market in Lahore, Pakistan.

### Collection of wastewater and physicochemical examination of industrial effluent

Textile effluent was collected from the industrial area of Kot Lakhpat, Lahore, Pakistan, and stored at 4°C until use. The physical parameters, such as color, pH, and temperature, of the sample were recorded on-site, while BOD, COD, total dissolved solids (TDS), and total suspended solids (TSS) were measured as described by Siddique et al. (2025).

### Screening and characterization of dye-decolorizing bacteria

Strains of bacteria capable of growing on azo dyes (SB, SY, SR) were isolated by enrichment. 20 mL of textile effluent was added to 80 mL of MSM containing 50 mg/L each of the reactive dyes, and the mixture was incubated at 37°C for 7 days. After incubation, serial dilutions were coated on MSM agar plates supplemented with 50 mg/L of each reactive dye. Plates were left to incubate at 37°C for 72 h. After 72 h, individual bacterial colonies were picked and purified by the sub-culturing technique (Fareed et al., 2022). Morphological characterization, followed by Gram staining, was performed to study the characteristics (Rima et al., 2022).

### Initial screening of dye-decolorizing bacteria in a shake flask using UV-Vis spectrometry

The cultures were cultured for 7 days after a loopful of each of the six colonies was individually inoculated into MSM supplemented with 50 mg/L of each reactive dye. The biomass and supernatant were then separated by centrifuging the samples for 1 min at 13,000 rpm. The supernatant was used to determine the concentration of azo dyes by measuring absorbance at 600 nm with a spectrophotometer (Sreedharan et al., 2021). The dye decolorization rate (%) was measured using the following formula.


%ofdyedecolorization

$$=\frac{\mathrm{Initial}\,\mathrm{absorbance}-\mathrm{final}\,\mathrm{absorbance}}{\mathrm{initial}\,\mathrm{absorbance}}\times 100$$

### Evaluation of dye tolerance in bacterial strains at varying concentrations of azo dyes

The dye tolerance of six isolated strains was assessed by exposing them to azo dyes at increasing concentrations (50, 100, 150, 200, and 250 mg/L). The survival of bacteria under dye-induced stress conditions was monitored by incubating cultures at 37°C for 96 h. Uninoculated flasks containing the same dye concentrations served as controls.

### Identification through 16S rRNA gene sequencing

Using conventional primers 27F and 1492R for PCR amplification of the 16S rRNA gene, the top-performing bacterial strain, SZ1, was found. The NCBI BLAST program^1^ was used to compare the acquired sequence with sequences in the NCBI GenBank database. The Multiple Alignment Fast Fourier Transform (MAFFT) at EMBL-EBI^2^ was used to combine and align query and reference sequences. MEGA XII (Molecular Evolutionary Genetics Analysis-XII) was used to create a phylogenetic tree using the obtained alignment and tree files. The tree was visualized and edited using the iTOL web server (Interactive Tree of Life)^3^ (Bhayana et al., 2025).

### Growth optimization of SZ1 at various physicochemical parameters

The strain SZ1 was examined under varying set of conditions (ranges from 1, 3, 5, 7, 9, 11, and 13), incubation temperature (ranges from 30 to 40°C), incubation days (1, 3, 5, and 7) and inoculum size (1, 2, 3, and 4%) in order to maximize the decolorization potential (Khan et al., 2025).

### Development of floating treatment wetlands

#### Establishment of reactive dye-enriched industrial wastewater

Wastewater containing reactive dye was synthetically prepared at a dye concentration of 100 ppm.

#### Plant

*Typha domingensis* (southern cattail) was harvested from Minhala village near the Wahga Border in Lahore. It was selected as it is native and adaptable to diverse environmental conditions, and has been reported to enhance the removal of dyes and other organic pollutants.

#### Bacterial inoculum preparation

**FIGURE 1:**
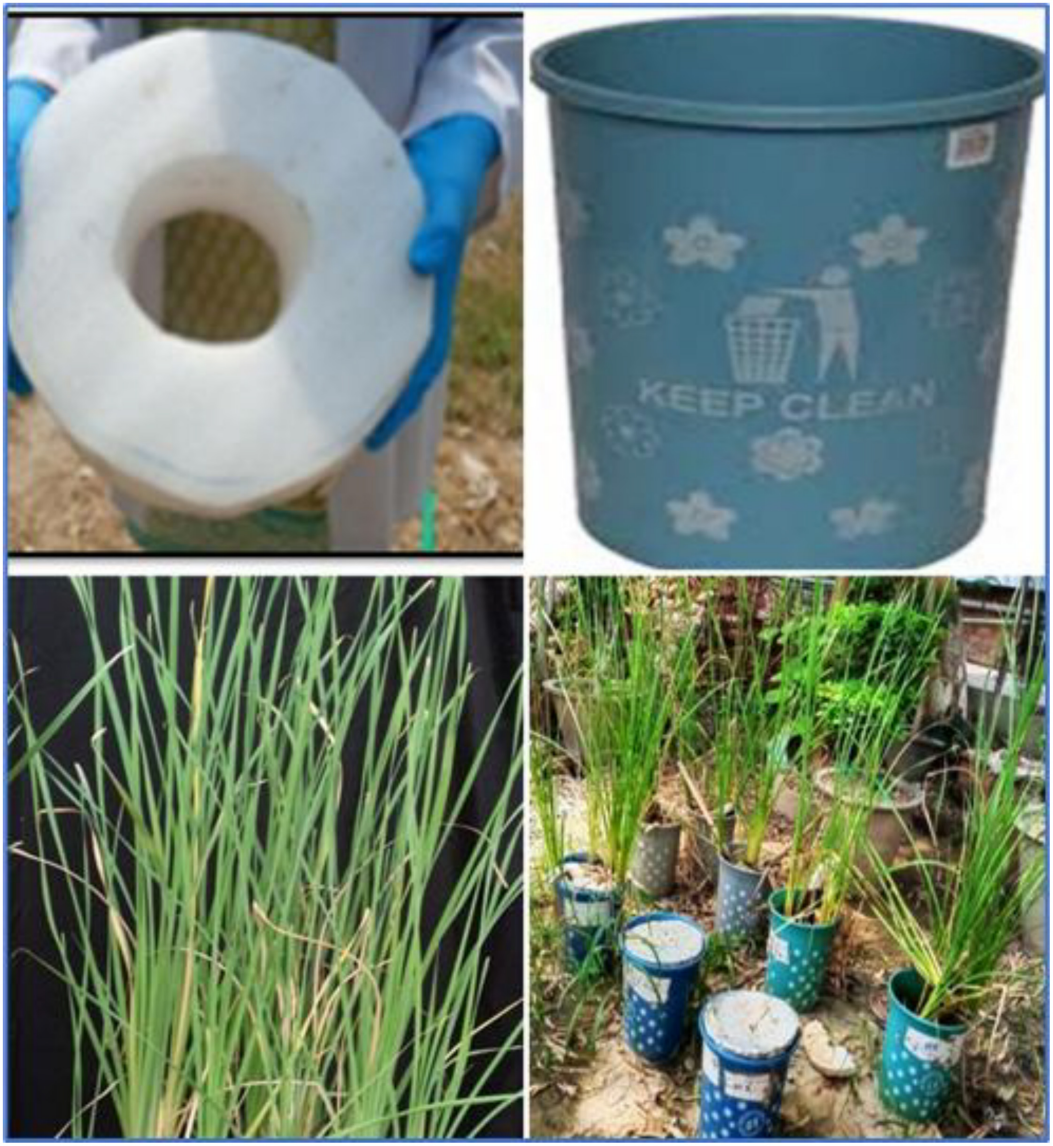
Schematic representation of the FTW setup for azo dyes removal.

A polystyrene sheet was used to create 36 FTW microcosms in total. Five healthy *T. domingensis* seedlings could fit in the hole made in the middle of the sheet after it was cut in a circle (Figure 1). This setup was placed on top of the water tanks containing 5 L of uncontaminated water. The plants were allowed to grow under uncontaminated water conditions, which were subsequently substituted with reactive dyes after 15 days. The experiment was run in triplicate. The details are as *Control 1 (C)*: Water contaminated with dye SR; *Control 2 (C)*: Water contaminated with dye SY; *Control 3 (C)*: Water contaminated with dye SB; *Treatment (T1)*: Water contaminated with SR and SZ1; *Treatment (T2)*: Water contaminated with SY and SZ1; *Treatment (T3)*: Water contaminated with SB and SZ1; *Treatment (T4)*: Water contaminated with dye SR and *T. domingensis*; *Treatment (T5)*: Water contaminated with dye SY and *T. domingensis*; *Treatment (T6)*: Water contaminated with dye SB and *T. domingensis*; *Treatment (T7)*: Water contaminated with dye SR, *T. domingensis* and SZ1; *Treatment (T8)*: Water contaminated with dye SY, *T. domingensis* and SZ1; *Treatment (T9)*: Water contaminated with dye SB, *T. domingensis* and SZ1.

#### Determination of plant growth and analysis of residual reactive dye concentrations in water

The shoot and root lengths of *T. domingensis* were measured after 90 days to assess the effects of reactive dye toxicity and bacterial inoculation on plant growth. The effluent collected after on-site treatment was evaluated for dye degradation (%) using a UV-Visible spectrophotometer, as described previously.

#### Phytotoxicity assay

Before and after treatment in FTWs, the toxicity of azo dyes to Triticum aestivum seeds was evaluated using a phytotoxicity test. After soaking in 0.1% HgCl_2_ for 2 min to surface-sterilize each seed, sterile distilled water was used to rinse them all. The seeds (*n* = 20) were placed in autoclaved Petri plates coated with blotting paper. For 5 days, 2 mL of tap water, dye-contaminated water (100 ppm), and treated water were added to petri plates. After 5 days, seed germination %, shoot, and root length were measured.

#### Statistical analysis

Analysis of variance (ANOVA) was utilized to evaluate significant differences between treatments after data were processed using SPSS (SPSS Inc., Chicago, IL, United States). Statistical significance was determined using a *p*-value of < 0.05. A *post-hoc* Tukey’s HSD test was subsequently conducted at α = 0.05 to assess significant differences.

## Results and discussion

### Isolation and *in vitro* dye decolorization assay

**FIGURE 2:**
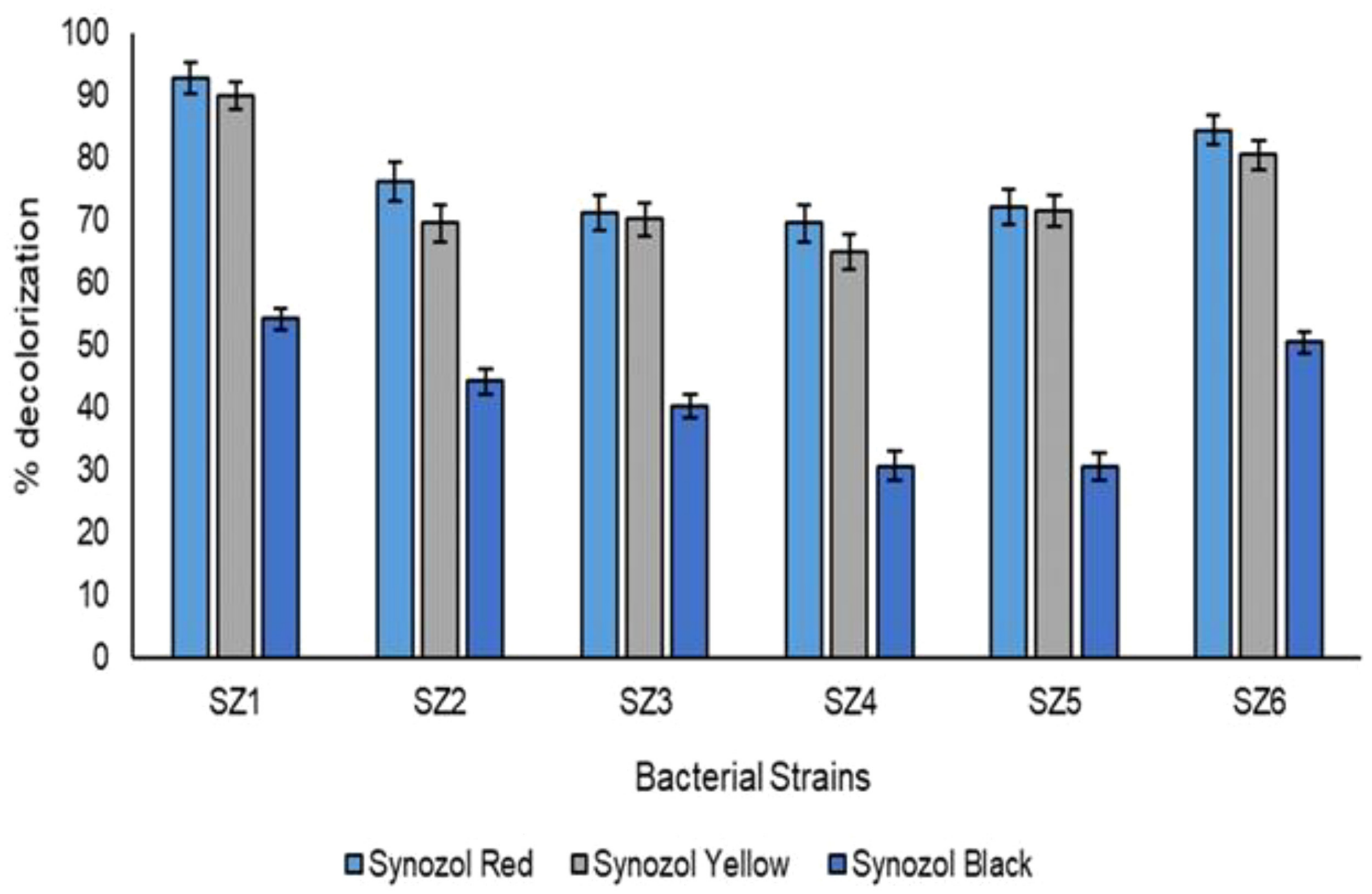
The comparative decolorization of azo dyes by isolated bacterial strains is shown in this figure. A total of six bacterial isolates (SZ1, SZ2, SZ3, SZ4, SZ5, and SZ6) were tested for their dye decolorization ability of SR, SY, and SB. The mean of 3 independent replicates and standard deviation are shown with the error bars.

The physicochemical characteristics were determined by analyzing the wastewater sample. The wastewater exhibited a pH of 6.8, a COD of 1,171 mg/L, and a BOD of 130 mg/L, indicating a considerable organic pollution load. Six isolated bacteria were obtained from the textile effluent samples and were named SZ1 through SZ6. Only two isolates (SZ1 and SZ6) were found to show visible color zone around their colonies by initial screening, showing some dye-decolorizing capability. These strains were also assessed in the decolorization of these dyes by shake flask assay. ANOVA revealed significant effects of both dye type and bacterial strains on decolorization efficiency (*p* < 0.05). Among all strains, SZ1 and SZ6 showed 92–84% decolorization of SR, 89.96–80% decolorization of SY, and 54–50% decolorization of SB dyes, respectively (Figure 2). A consistent pattern was observed across decolorization for all isolates, i.e., monoazo (SR and SY) > diazo dyes (SB). Thus, decolorization is significantly linked to both dye complexity and bacterial strain. The capacity of different organisms to degrade dyes varies with the dye type, the organism, and numerous environmental factors, such as temperature and pH (Henagamage and Peries, 2023; Ali, 2010). The current findings are in line with research conducted by Emadi et al. and Khan et al. (2025), which found that diazo dyes (AB-113: acid blue 113) decolorized more slowly than mono-azo dyes (MR: methyl red and MO: methyl orange).

### Molecular identification and phylogenetic analysis

**FIGURE 3:**
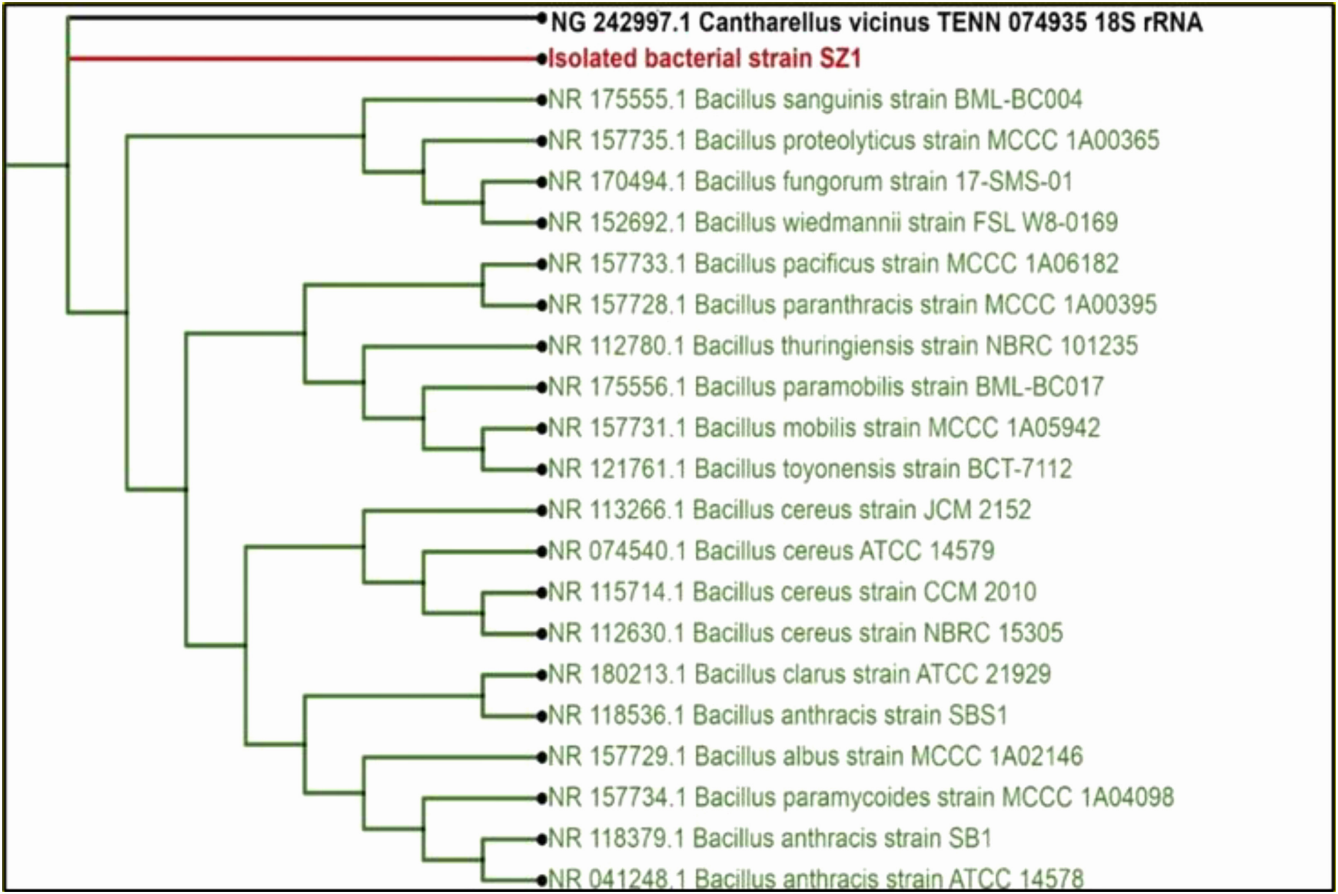
Phylogenetic analysis and taxonomic position of bacterial strain SZ1 determined by sequences of the 16s rRNA gene. Cantharellus vicinus TENN was chosen as an Outgroup. Phylogenetic tree was inferred using the maximum-likelihood approach with 1,000 replicates of bootstraps. The tree was generated using MEGA XII and designed by iTOL v6.

The best-performing strain, SZ1, was identified by nucleotide BLAST (BLASTn) with a cutoff of > 95%. BLAST analysis revealed that the query sequence exhibits 100% similarity with *Bacillus thuringensis*, *Bacillus paramycoides*, *Bacillus anthracis*, and *Bacillus albus.* Sequences were then extracted from the NCBI GenBank database in FASTA format. A phylogenetic tree was then built using these sequences (Figure 3). Phylogenetic analysis revealed that strain SZ1 clustered with *Bacillus* genus, however, the 100% sequence identity observed across the four species in the sequenced region excluded species-level discrimination (Albayrak and Uluçay, 2026). Consequently, strain SZ1 was tentatively identified as *Bacillus* sp. (GenBank accession number: PZ493129). Further molecular techniques, such whole-genome sequencing (WGS) or multilocus sequence typing (MLST), would be necessary for species-level categorization (Li et al., 2020; Sraphet and Javadi, 2025). Congo Red, Reactive Black 5, Reactive Green 19, Reactive Red 120, and Reactive Blue 4 were among the azo dyes that *Bacillus* subtilis CKCC decolorized. Additionally, under anaerobic circumstances, *Bacillus* cohnii, *Bacillus* sp. YZU1, and *Bacillus* pumilus were reported to decolorize direct dyes (Fareed et al., 2022; Seyedi et al., 2020). Thus, *Bacillus* spp. are widely prevalent in dye-contaminated environments (Zhuang et al., 2023; Sun et al., 2025) because of their ability to tolerate high concentrations of reactive azo dyes, diverse enzymatic activity (azoreductase and ligninolytic enzymes such as laccase, manganese peroxidase and lignin peroxidase), and adaptability, which makes them key members of microbial communities in textile effluent and wastewater treatment systems.

### Influence of dye concentration on azo dye decolorization

**FIGURE 4:**
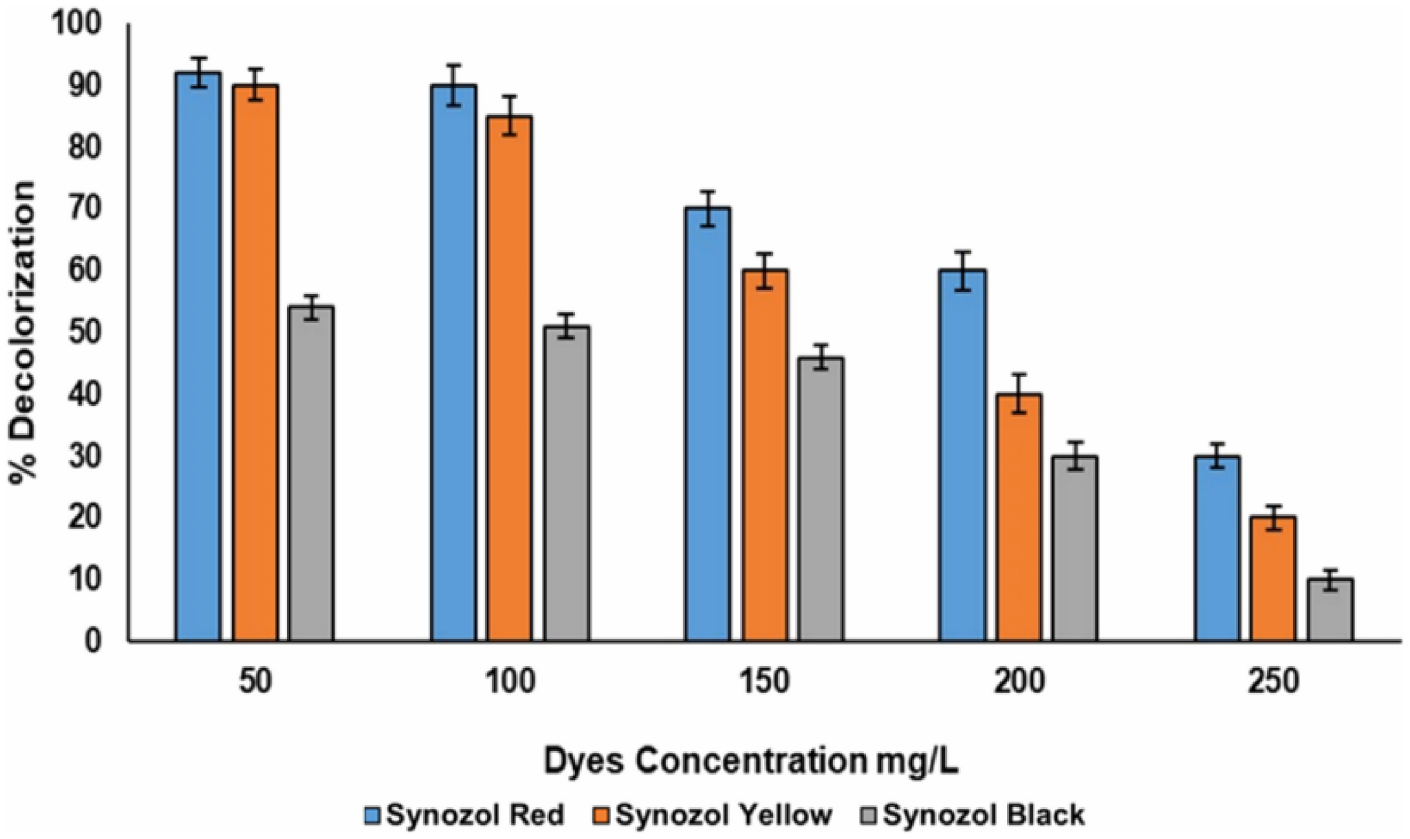
Effect of varying concentrations of azo dyes (50, 100, 150, 200, and 250 mg/L) on the percentage decolorization of dyes by *Bacillus* sp. SZ1.

The maximum decolorization was found at 50 mg/L and 100 mg/L dye concentration for SR dye (∼90–92%), SY dye (∼85–89%) and SB dye (∼51–54%). Further reductions were noted at 150 mg/L (SR 70%, SY 60%, SB 46%), 200 mg/L (SR 60%, SY 40%, SB 30%), and 250 mg/L (SR 30%, SY 20%, SB 10%) (Figure 4). The dye concentration significantly affected the decolorization efficiency (*p* < 0.05). The highest decolorization was recorded at 50 mg/L, followed by 100, 150, 200, and 250 mg/L, indicating a progressive decrease in decolorization with increasing dye concentration. The reduction in the decolorization percentage might be due to a higher concentration of dye molecules blocking enzyme active sites or to dye toxicity. Similar studies have also reported that lower dye concentrations, such as 100 mg/L, are optimal for decolorization. The literature indicates that lower dye concentrations reduce the toxic load on bacterial cells, thereby ensuring optimal growth and activity (Alsukaibi, 2022). In contrast, higher dye concentrations limit decolorization by inhibiting microbial metabolism and enzyme activity through toxic effects.

### Optimization of dye degradation by various physicochemical parameters

**FIGURE 5:**
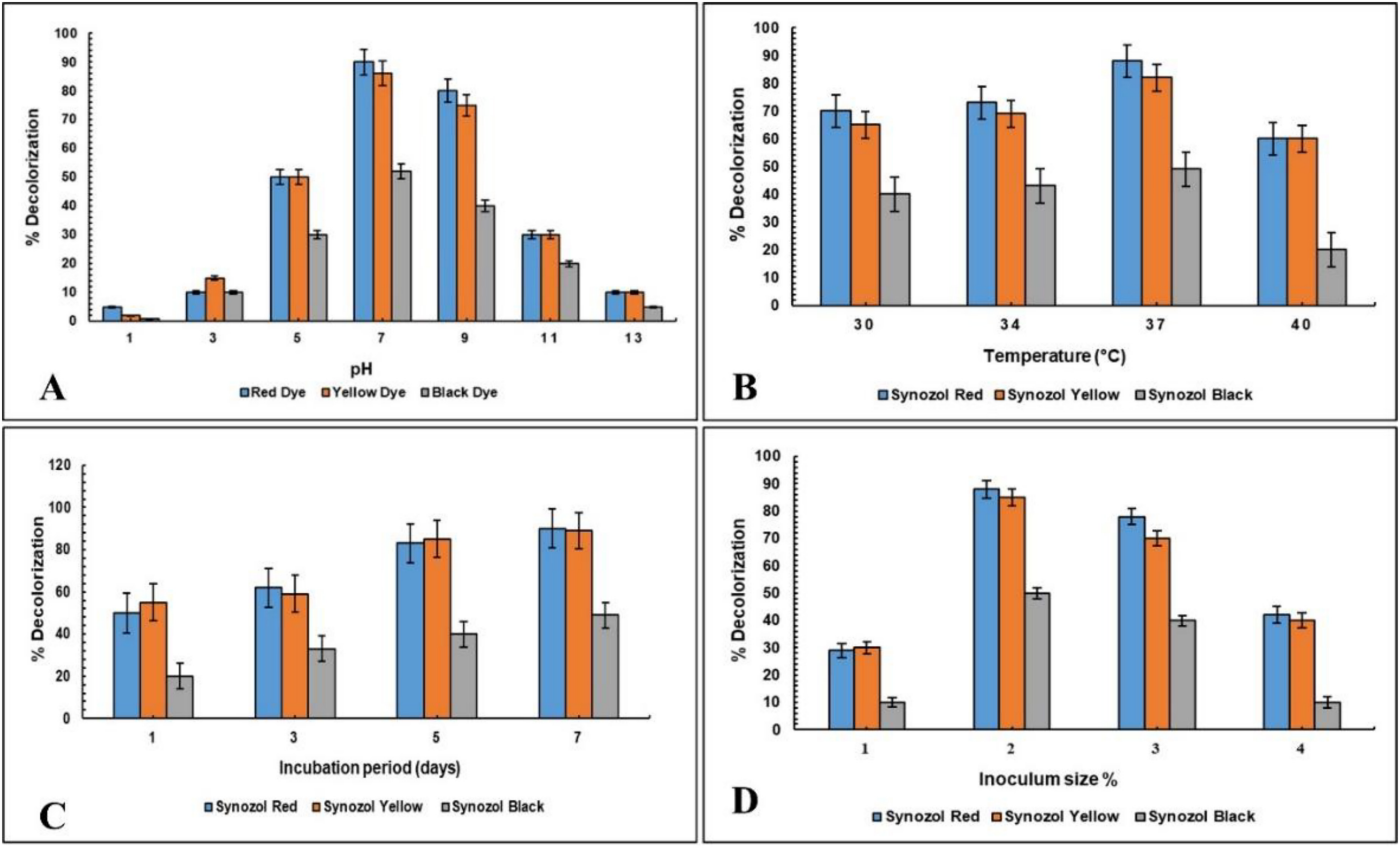
Graph represents the percentage decolorization consistent to varying physicochemical parameters for bacterial strain SZ1: **(A)** pH, **(B)** temperature, **(C)** incubation period, and **(D)** inoculum size.

The results indicate that maximum dye decolorization occurred at pH 7, a 2% inoculum size, a 7-day incubation period, and 37°C (Figure 5). At pH 7, the decolorization percentages were approximately 92% for SY dye, 89% for SR dye, and 54% for SB dye (Figure 5a). With a 2% inoculum size, red and yellow dyes achieved around 88–85%, while black dye achieved a 50% decolorization. With a 7-day incubation period, yellow and red achieved maximum decolorization (∼90 and 89%, respectively), while black dye reached a maximum of 49%. At 37°C, red and yellow dyes achieved removal rates of around 88 and 82%, while black remained at about 49% (Figure 5b). Dye decolorization was significantly lower at extreme pH values, at very low or high inoculum sizes (1 and 4%), and at short incubation periods (Figure 5c), indicating that dye removal depends on various optimal physicochemical and biological factors. This is probably due to the fact that the values of pH 7 are optimum for *Bacillus* sp., and any extremes of pH can inactivate several enzymes that break down dyes, including azoreductases, peroxidases, and laccases (Khan et al., 2014). The microbial biomass is enough to decolorize azo dyes but not compete for nutrients in the medium if the inoculum size is kept at 2% (Figure 5d). The 7-day incubation period allows bacteria to grow, express enzymes and completely break down the dye molecules, especially complex ones like azo bonds. Finally, the 37°C temperature is consistent with the optimal range for *Bacillus* spp. to maximize enzyme activity, membrane fluidity, and metabolic processes (Mishra et al., 2022; Ikram et al., 2022). During the biodegradation of contaminants, various physicochemical factors (pH, temperature, carbon source, dye concentration, and incubation period) directly influence the decolorizing capacity of microorganisms (Khan et al., 2025).

### Bacterial inoculation’s impact on plant development and dye decolorization in FTWs

To gauge the impact of the dye and bacterial inoculation on plant development, the root and shoot lengths of *T. domingensis* were measured at the conclusion of the experiment.

**FIGURE 6:**
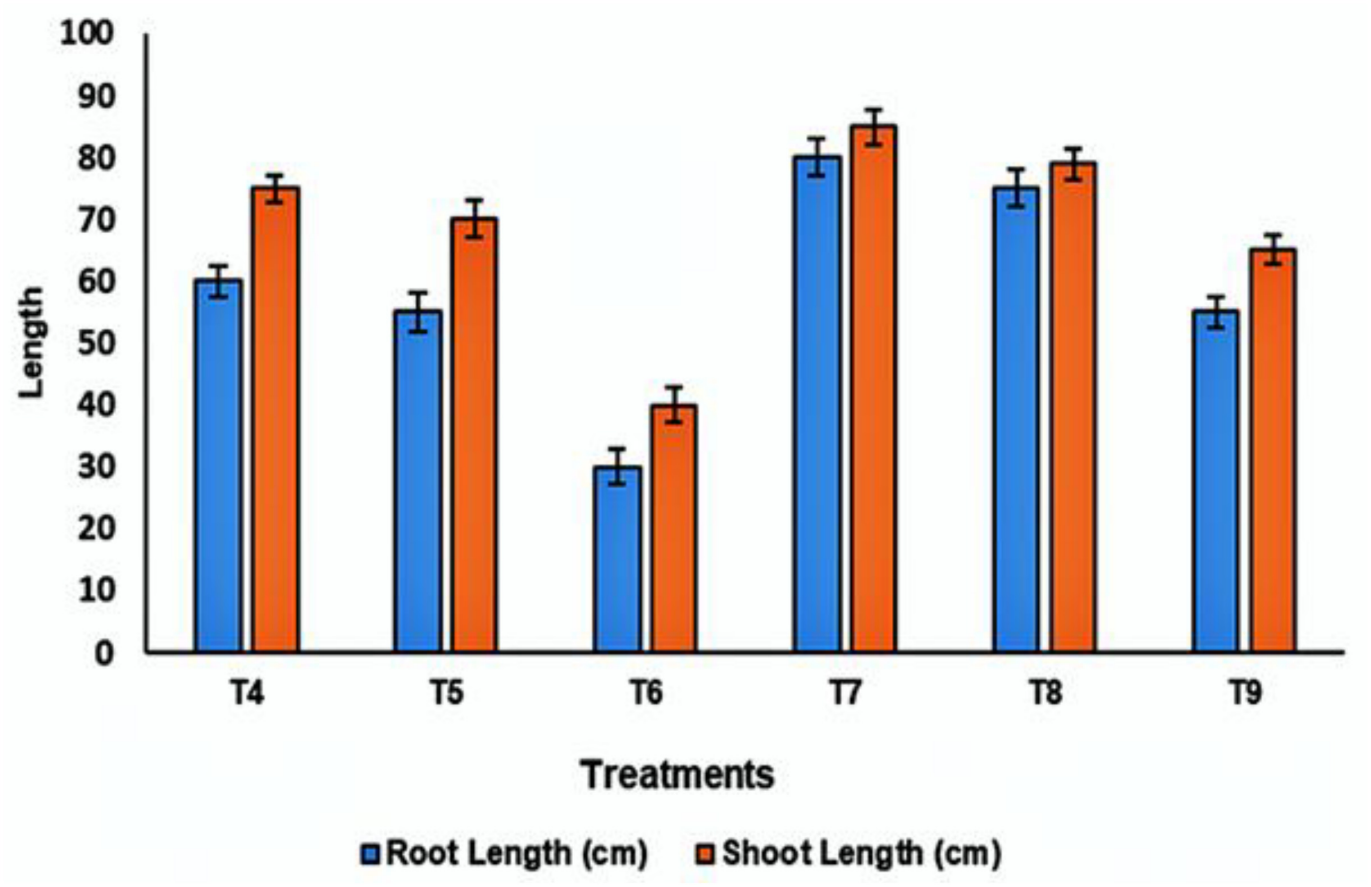
Effect of different treatments on root and shoot length of *T. domingensis* vegetated FTWs. Each value is the mean of three replicates, and error bars represent the standard deviation.

Dye-contaminated water has a significant effect (*p* < 0.05) on the plant growth, but bacterial inoculation enhanced root length (33%) and shoot length (13%) of the plant (Figure 6). T7 and T8, which combined the utilization of plants and bacteria, showed the greatest plant growth as compared to the other FTW-designed treatments. Despite the toxicity of dyes, these findings support the positive benefits of bacterial inoculation on plant development. Microorganisms play an essential role in plant growth and in the removal of toxic contaminants, thereby lessening the negative impact on the plant. This could be in response to the enzymatic activities that occur jointly in plants and bacteria to break down the dyes into simpler products. Additionally, under stress, numerous biochemical and molecular pathways are activated to enhance biological activities, such as phytohormone production and ACC deaminase activity (Tara et al., 2019).

**FIGURE 7:**
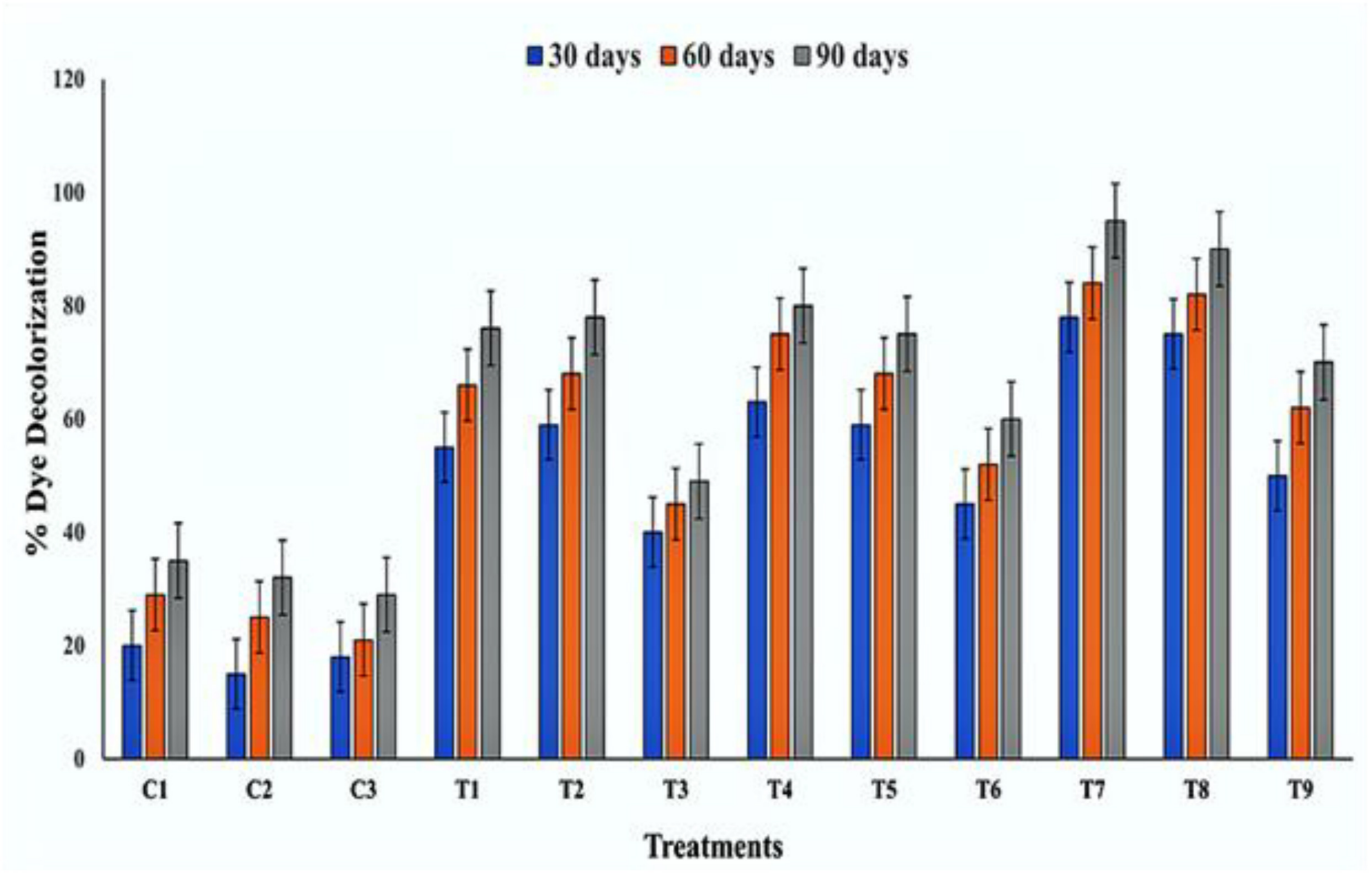
Influence of various treatments on the percentage of dye decolorization in a greenhouse experiment. The provided values represent the average of three replicates, with the error bar representing the standard deviation.

The percentage of dye decolorization of various treatments (T1–T9) after 30, 60, and 90 days is shown in Figure 7. The activity was found to be time-dependent as a consistent trend of increasing decolorization efficiency with time was observed for all the treatments. The treatments resulted in significant decolorization with the highest rate recorded in T7, nearly 90% at 90 days, and T8 also performed well with more than 80% decoloration. The findings are in line with those of previous studies that found bioaugmented FTWs more effective for dye decolorization than non-bioaugmented ones, attributed to the synergistic effect of plant roots and their associated microorganisms (Rendón-Castrillón et al., 2024; Sahreen and Mukhtar, 2023).

### Phytotoxicity assessment of treated water

A seed germination experiment was used to assess the harmful effects of azo dyes (SR, SY, and SB) treated in FTWs. Triticum aestivum seeds treated with water vs. dye-enriched water were compared in terms of seed germination, shoot, and root lengths (Table 1). According to the findings of the phytotoxicity bioassay, the FTW treatments created in this study considerably decreased the toxicity of the dye and its broken-down metabolites. The results align with those of earlier studies that demonstrated that treated wastewater from bioaugmented FTWs was less phytotoxic and resulted in significantly higher germination and growth of wheat seedlings (Rashid et al., 2024; Singh et al., 2024).

**TABLE 1:** Shows the effect of dyes, *Bacillus* sp., *Typha domingensis* and their synergistic interaction on the growth of Triticum aestivum.

Treatments	% Germination	Shoot length (cm)	Root length (cm)
Tap water	95	9.1 ± 0.81	5.1 ± 0.84
C1	35	2.3 ± 0.04	1.14 ± 0.04
C2	30	2.1 ± 0.20	1.43 ± 0.16
C3	20	1.9 ± 0.09	0.9 ± 0.2
T-1	85	7.16 ± 1.02	3.9 ± 0.64
T-2	80	6.83 ± 1.43	2.5 ± 0.4
T-3	50	5.33 ± 0.47	2.9 ± 0.41
T-4	75	6.93 ± 0.09	3.26 ± 0.71
T-5	80	6.66 ± 0.47	3.16 ± 1.31
T-6	65	5.5 ± 1.47	2.17 ± 1.02
T-7	90	8.9 ± 0.7	3.75 ± 0.40
T-8	85	8.6 ± 0.61	3.46 ± 0.62
T-9	70	6.06 ± 0.41	2.56 ± 0.41

## Conclusion

The results showed that bioaugmented FTWs with T. domingensis and *Bacillus* sp. were effective and environmentally friendly approach for the treatment of textile effluent contaminated with azo-dye. Under optimal physicochemical conditions, the strain SZ1 of the genus *Bacillus*, out of six bacterial isolates, showed the highest ability in dye decolorization, especially in the removal of SR and SY. SZ1 was highly effective in dye removal, as high as 95% in FTWs, and better in plant growth parameters, showing decreased toxicity. This environmentally friendly, cost-effective technology is an effective substitute to traditional wastewater treatment methods for the treatment of industrial wastewater. This system is still to be validated at field scale and to assess its performance in long-term conditions in real textile effluents.

## Data Availability

The original contributions presented in this study are included in the article/supplementary material, further inquiries can be directed to the corresponding author.

## Footnotes

1
https://blast.ncbi.nlm.nih.gov/Blast.cgi

2
https://www.ebi.ac.uk/jdispatcher/msa/mafft

3
https://itol.embl.de/
